# Huge mesenteric desmoid-type fibromatosis with unusual presentation: A case report

**DOI:** 10.1016/j.amsu.2022.103741

**Published:** 2022-05-10

**Authors:** Mohamed Hajri, Ghofrane Talbi, Wael Ferjaoui, Aziz Atallah, Sana Ben Slama, Hafedh Mestiri, Rached Bayar

**Affiliations:** a Department of General Surgery, Mongi Slim University Hospital, Tunis, Tunisia; b Department of Pathology, Mongi Slim University Hospital, Tunis, Tunisia; c Faculty of Medicine of Tunis, Tunis El Manar University, Tunis, Tunisia

**Keywords:** Desmoid tumors, Fibromatosis, Peritonitis

## Abstract

**Introduction:**

Desmoid-type fibromatosis, also known as desmoid tumors, are rare fibroblastic neoplasms that account for less than 3% of all soft tissue tumors. Although they are benign neoplasms without metastatic potential, they are known to be locally aggressive and may invade adjacent structures leading to fatal complications.

**Case presentation:**

We describe the case of a 26-year-old woman who presenting with the clinical picture of acute peritonitis. Emergency surgery was performed and a large poorly-circumscribed heterogeneous tumor was found, occupying the jejunum mesentery and infiltrating the jejunal wall causing its perforation into the abdominal cavity. En bloc resection of the tumor and the involved jejunum was performed. Histology and immunohistochemistry confirmed it to be mesenteric desmoid-type fibromatosis. The postoperative course was uneventful and the patient had no evidence of recurrence 18 months after tumor resection.

**Conclusions:**

Mesenteric desmoid-type fibromatosis is a rare condition with insidious growth and locally aggressive behavior. Serious complications such as bowel perforation are rare but possible, as shown in our presentation. Complete surgical resection is the first-line treatment bur high recurrence rates remain problematic.

## Highlights

• Desmoid-typefibromatosis are rare fibroblastic neoplasms that account for less than 3% of all soft tissue tumors.
• We reported a rare case of diffuse peritonitis due to sporadic mesenteric desmoid-type fibromatosis with aggressive proliferation and consecutive intestinal perforation. Only a few similar cases have been reported in the literature.
• This case report an unusual presentation of mesenteric desmoid.

## 1 Introduction

Desmoidtumors, also known as fibromatosis, are benign fibroblastic neoplasms that account for less than 3% of all soft tissue tumors [1]. The most occurring locations are the limbs, the abdominal wall and rarely the abdominal cavity, particularly the mesentery. They can occur sporadically or in the context of congenital syndromes such as familial adenomatous polyposis (FAP) [2]. Although demoid tumors are benign neoplasms without metastatic potential, they are known to be locally aggressive and may invade adjacent structures leading to fatal complications [3]. We report hereby a rare case of a mesenteric desmoid-type fibromatosis presenting with intestinal perforation and acute diffuse peritonitis.

This case follows 2020 SCARE guidelines for reporting of cases in surgery [4].

## 2 Case presentation

A 26-year-old woman presented to the emergency department with a 24-h history of increasing abdominal pain with nausea and vomiting. Her past medical history was unremarkable and there was no notable family history. She had no Drug History and Allergies. She reported a change in bowel habits since 3 months.

Clinically, she was awake and conscious, with a temperature at 37.4°, blood pressure at 11/06 cmHg, heart rate at 110/min and respiratory rate of 18 breaths/min.

Abdominal examination revealed diffuse tenderness with a mobile mass on palpation in the mid-abdomen.

Laboratory studies showed a normal white blood cell count of 5.77 × 10^9^/L (normal range, 3.50–9.50 × 10^9^/L), elevated blood levels for C-reactive protein (203 mg/L; normal range, 0–10 mg/L) and normal kidney and liver tests.

**Fig. 1:**
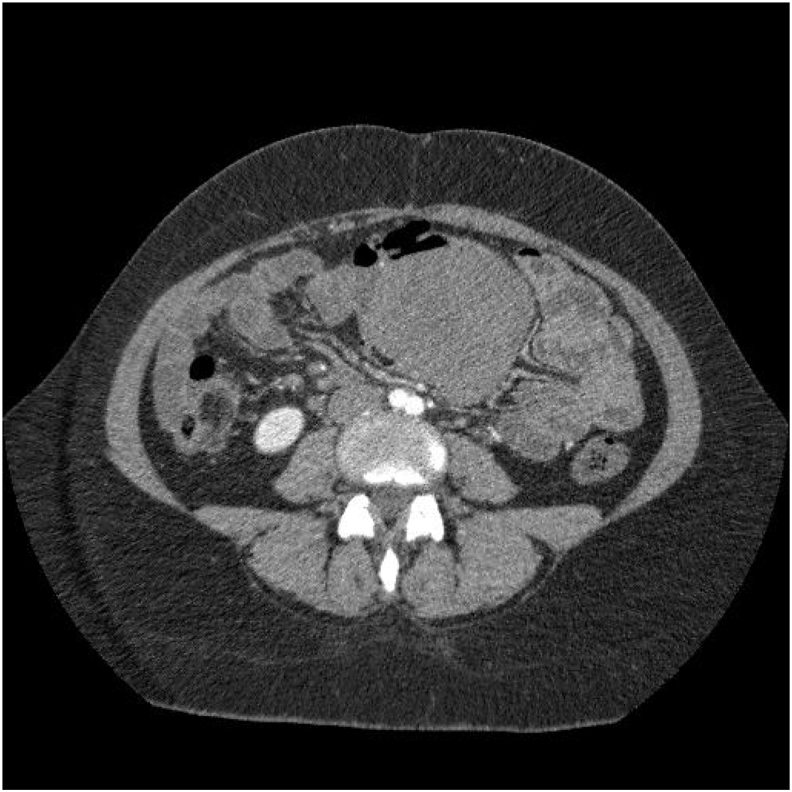
Abdominal computed tomography showing the 23 × 11 × 10 cm mesenteric desmoid tumor with small intestine compression and free intraperitoneal air.

Computed tomography (CT) demonstrated a 23 × 11 × 10 cm intra-abdominal mass with small intestine compression and free intraperitoneal air and ascites (Fig. 1).

**Fig. 2:**
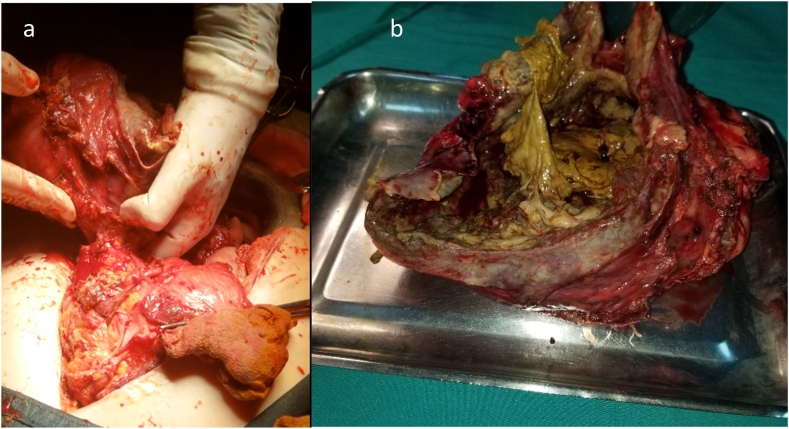
(a) Intraoperative findings showing a large poorly-circumscribed mesenteric desmoid tumor infiltrating the jejunal wall causing its perforation. (b) The resected specimen.

Emergency surgery was performed by an associate professor in general surgery, under general anesthesia with the patient in dorsal decubitus position. Intraoperatively, a large poorly-circumscribed heterogeneous tumor was found, occupying the jejunum mesentery and infiltrating the jejunal wall (Fig. 2) causing its perforation into the abdominal cavity. The above imaging and intraoperative findings suggested a GIST invading the small bowel. En bloc resection of the tumor with a 30 cm segment of the small bowel was performed and a double-barrel ileostomy was carried out. The postoperative course was uneventful.

**Fig. 3:**
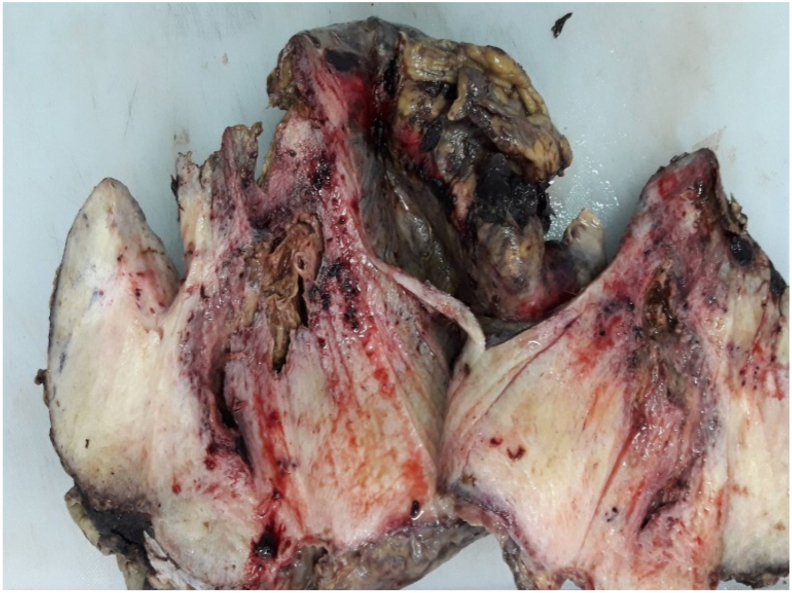
Gross examination showing a solid mass poorly circumscribed, firm, whorled and white cut surface.

Gross examination showed a solid mass poorly-circumscribed, firm, whorled and white cut surface (Fig. 3).

**Fig. 4:**
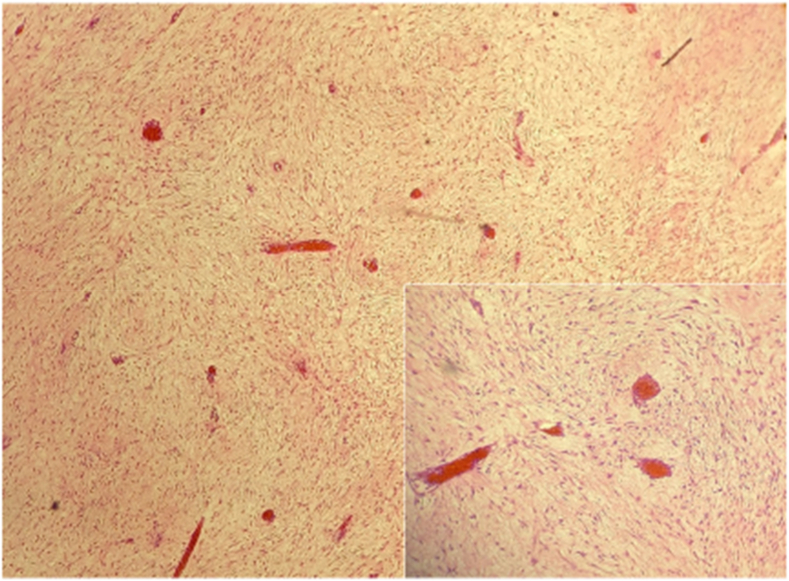
Microscopic examination showing proliferation of long sweeping fibroblasts and myofibroblasts (hematoxylin-eosin: HEx100).Cartridge: Cells demonstrate eosinophilic cytoplasm without cytologicatypia, blood vessels with perivascular oedema (hematoxylin-eosin: HEx200).

Histological examination showed fibroblastic and myofibroblastic proliferation with many bundles of spindle-shaped cells.In immunohistochemistry, it was positive for B-catenin, Desmin and SMA, and negative for CD117, CD34 and S100 protein (Fig. 4), supporting the diagnosis of desmoid-type fibromatosis.

Since postoperative total colonoscopy found no polyposis, the tumor was considered to be sporadic.

She recovered well and restoration of bowel continuity was performed two months after surgery. She had no evidence of recurrence 18 months after tumor resection.

## 3 Discussion

Desmoid-type fibromatosis (DF), also known as desmoid tumors or aggressive fibromatosis, are monoclonal fibroblastic proliferations arising in musculoaponeurotic structures [5].

They are reported to account forless than 3% of all soft tissue tumors and less than 0.03% of all neoplasms [6].

Although DF are benign neoplasms with no potential for metastasis or dedifferentiation, they exhibit a locally aggressive infiltrative behavior [5].

It can present sporadically or as a part of congenital syndromes such as FAP or Gardner's syndrome. Thus, colonoscopy is recommended in DF patients in order to detect polyps [7].

Female gender, estrogen exposure, previous abdominal surgery and trauma have been found to be associated with the occurrence of these tumors [8,9].

Our patient have not experienced prior surgery or trauma, and total colonoscopy did not detect polyposis, thus DF was considered to be sporadic.

While intra-abdominal site is very common in FAP-related cases, it is very rare in sporadic setting, accounting for less than 5%–10% of cases, and the mesentery is the most occurring intra-abdominal location [10].

The diagnosis of mesenteric desmoid-type fibromatosis (MDF) is usually delayed due to its insidious development as well as its non-specific clinical signs [11].

Given the locally aggressiveness of the disease, this can lead to several life-threatening complications caused by adjacent tissues invasion including bowel obstructions, perforations, and ischaemia [12].

We reported a rare case of diffuse peritonitis due to sporadic MDF with aggressive proliferation and consecutive intestinal perforation. Only a few similar cases have been reported in the literature [[12], [13], [14], [15]] [[12], [13], [14], [15]] [[12], [13], [14], [15]].

A recent Italian study reported 72 patients treated at IstitutoNazionaledeiTumori (INT) in Milano from 2005 to 2020 for intra-abdominal DF. Among these patients, only 5 presented with bowel perforation. The DF was of mesenteric origin in all 5 cases [12].

Differential diagnoses of MF include essentially GISTs, fibrosarcomas, lymphomas and carcinoid tumors.

MDF may occasionally mimic GISTs arising from the mesentery or peritoneum because of their morphological similarities [16,17]. Differentiating MDF from GISTs is important due to the differences in treatment [16,18].

Histologically, both DF and GISTs are characterized by a proliferation of spindle-shaped cells, so differentiation using routine hematoxylin and eosin staining is difficult. However, distinction between these tumors is based on immunohistochemical and molecular analytical techniques. While GISTs are known to show positive stainings for CD117, CD34, DOG 1 and PDGFRA, DF are characterized by positive beta-catenin expression and lack of CD34 expression. Hence, staining for beta-catenin and CD34 is recommended whenever there is diagnostic doubt between MF and GISTs [16].

Surgery is the mainstay treatment for MDF, and complete surgical resection is recommended when technically feasible. However, despite complete resection with negative microscopic margins, local recurrence rate is high and may be to 40%–70% [19]. Associated congenital syndromes such as FAP or Gardner's syndrome are seemed to be risk factors of recurrence [20,21]. It has been reported that DF recurred in 90% of patients with FAP and 11% of patients without FAP [22].

Besides surgery, the DF management involves multiple other approaches. Radiotherapy can play a role in unresectable cases, recurrent tumors or after incomplete resection.

Janssen et al. reported a meta-analysis including 1295 patients treated for DF. Adjuvant radiotherapy appeared to reduce the risk of recurrence after incomplete surgical resection, particularly in patients with recurrent tumors. However, it had no detectable benefit on recurrence after complete surgical resection with negative margins [23]. In a systematic review reported by Seinen et al., adjuvant radiotherapy with a combined dose of ≥50 Gy showed a significant advantage over surgery alone [24]. Nevertheless, the use of radiotherapy for the intra-abdominal DF is limited because of its bowel toxicity and consecutive enteritis.

Some systematic treatment options such as non-steroidal anti-inflammatory drugs, tyrosine kinase inhibitors, cytotoxic chemotherapy (vinblastine, doxorubicin) and antihormonal therapies have been reported to be effective [25,26]y. However, additional studies are needed to develop the optimal strategy for DF treatment.

In our case, we managed an emergent case of intestinal perforation due to huge infiltrative MDF. Emergency surgery was inevitable and complete resection of the tumor and a 30 cm of the small bowel was performed. Histology showed negative margins and no adjuvant therapy was carried out. Although the patient had no evidence of recurrence 18 months after surgery, further follow-up is mandatory.

## 4 Conclusion

MDF is a rare condition with insidious growth and locally aggressive behavior. Serious complications such as bowel perforation are rare but possible, as shown in our presentation. Complete surgical resection is the first-line treatment bur high recurrence rates remain problematic. Multidisciplinary care is always necessary and further studies and clinical trials are required to establish structured guidelines.

## Patient perspective

The procedure of surgery was explained to the patient with all advantages and possible complications. He agreed on the procedure an informed consent was taken from her.

## Funding

The author(s) received no financial support for the research, authorship and/or publication of this article.

## Ethics approval

Not applicable.

This work has been reported in line with the SCARE 2020 criteria.

## Consent

Written informed consent was obtained from the patient for publication of this case report and accompanying images. A copy of the written consent is available for review by the Editor-in-Chief of this journal on request.

## Author's contribution

Mohamed Hajri, Ghofrane Talbi,Wael Ferjaoui: Writing, review and editing of the manuscript.

Aziz Atallah, Sana Ben Slama: Contributed for diagnose and treatment of the patient.

Hafedh Mestiri, Rached Bayar: Review, Supervision and surgeons of the patient.

## Registration of research studies

1 Name of the registry:

2 Unique identifying number or registration ID:

3 Hyperlink to your specific registration (must be publicly accessible and will be checked):

## Guarantor

Wael Ferjaoui.

## Provenance and peer review

Not commissioned, externally peer reviewed.

## Declaration of competing interest

The authors declared no potential conflicts of interests with respect to research, authorship and/or publication of the article.
